# The Principles and Practice of Medicine

**Published:** 1844-04

**Authors:** 


Art. IV.—*The Principles and Practice of Medicine.* By John Elliotson, M.D., Cantab.; F.R.S., President of the Royal Medical and Chirurgical Society; President of the Phrenological Society; late Professor of the Principles and Practice of Medicine, and of Clinical Medicine, and Dean of the Faculty of Medicine, in University-College, London; late Senior Physician to the University-College-Hospital; Fellow of the Royal College of Physicians; formerly Physician to St. Thomas's Hospital, and President of the Royal Medical Society of Edinburgh. Edited by Nathaniel Rogers, M.D., Member, and late President, of the Hunterian Society of Edinburgh; and Corresponding Member of the Medico-Chirurgical Society of Dublin; and Alexander Cooper Lee. *First American, from the Second London Edition, greatly enlarged and improved. With notes and additions;* by Thomas Stewardson, M.D., Physician to the Pennsylvania Hospital. 8vo. 1 vol. pp. 1045. Carey & Hart. Philadelphia. 1844.

It is fortunate for the modern critic, that he is not compelled by the usages of his profession to graduate his reviews by the size, the length, the breadth, or the *weight* of the works which he is obliged from time to time to notice. Were the reverse the case, a long period would necessarily elapse before we should be permitted to drop the curtain between our readers and the volume which stands at the head of this article. Of the very numerous treatises on the Principles and Practice of Medicine with which the press has teemed during the last quarter of a century, that of Dr. Elliotson is not only one of the most ponderous, but, in many respects, one of the most singular that has fallen under our observation. Published in octavo form, bound in substantial sheep, and comprising upwards of one thousand closely printed pages, its weight, we are assured, is fully equal to that of the fœtus at the end of the seventh month. We do not mean by this to intimate that its birth was premature, or that it would have grown to a still more gigantic size, if time had been allowed the learned author to bring it out himself. Its title-page exhibits, it will be observed, in large and small type, not less than twenty-eight lines, interspersed with the names of four individuals, one of whom stands in the light of principal, the others in that of "aiders and abettors" of this extraordinary offspring of the medical press. The principal, to whom we owe the bone and sinew of the work, had no agency, it would seem, in sending it forth. The lectures which he delivered were copied, published in medical journals, and, attracting an uncommon share of attention, have been arranged and sent abroad by the three editors in their present form.

The volume, which is unquestionably one of great learning, is dedicated with perfect propriety to Lord Brougham, the noble patron of letters in Great Britain, and the only omission which one regrets to find, in contemplating its portly figure and *Falstaffish* physiognomy, is that it is not embellished by the likeness of the author, his commentators, and, especially, his American *appendiculus.* How so glaring a blunder could have been committed, considering the tact and taste of the editor at Philadelphia, is not easily understood. The omission certainly constitutes a defect which must strike every reader, and we therefore cannot forbear the expression of a hope, that it will be supplied in a second edition, an early call for which is rendered certain by the very valuable "notes and additions" of Dr. Stewardson. In that event we would respectfully suggest, that Dr. Stewardson, instead of placing himself, as he has done in the present instance, with characteristic modesty, at the foot of the page, "*locate*" himself in the *centre* of the frontispiece, with Dr. Elliotson above, Dr. Rogers on the right, and Mr. Lee to the left, with a wreath encircling the group, and resting upon the brow of the American editor. The latter gentleman is quite too modest. We insist upon his taking a more conspicuous position. His contributions to the volume before us entitle him to it. But if they do not, the fact of his having so generously taken it upon him to patronize the efforts of Dr. Elliotson, most unquestionably does. Dr. Elliotson, president of the phrenological Society of London, and late Professor of the Principles and Practice of Medicine in University-College, &c., is little known in this country, and the kindness of Dr. Stewardson in stepping forward to endorse his labors, cannot be too highly appreciated. The American profession, on this account, owes Dr. Stewardson a heavy debt of gratitude. But for this opportune service, Dr. Elliotson's Practice might have fallen still-born from the press, and thus would have been lost to our countrymen all the learning and experience which this great work embodies.

Seriously; we are no admirers of the *grafting* system so long and so much in vogue in this country. It is quite too much the fashion with us, to seize upon foreign books the moment they touch our shores, add a few meagre, if not worthless notes to them, stick a name into the title-page, and then strut abroad in large gilt letters upon the cover, with all the complacency of the jackdaw in his stolen feathers. Often, as in the present instance we suspect it will be decided, the size of the book is swelled, without any addition to its real value. Matter is infused into it which operates much like the water added by dishonest dealers to spirits. There is extension, but no increase of weight. The volume grows, but its additions are such as to cause its readers, in the bitterness of disappointment, to cry out "a *great book* is indeed a great evil." It is in such cases that we feel the force of the saying of the old poet, that "*the half is greater than the whole.*"

The volume to which Dr. Elliotson's name has been appended is a work, which could not have emanated from any but a great, original, and highly cultivated mind. It is replete with learning, and with the rich experience of a man who has seen much of disease, and is accustomed to think for himself. Every page bears the impress of a clear and untrammelled intellect, not free from prejudices, we dare say, and prone, perhaps, to be dazzled and carried away by novelties of a certain character. But it is a mind of so much vigor, so capable of enlarged views, and so frank and fearless in the adoption and advocacy of what is felt to be the truth, that one readily overlooks the few aberrations into which it may have been betrayed. In looking through the practical and judicious pages of this elaborate volume, one is disposed to forget that its gifted author gave himself up to the delusions of animal magnetism; and if any one should take the volume up with prejudices against it on that account, which we confess was our own case, they must be very perverse prejudices if they do not soon yield to the strong good sense in which it abounds.

Of course, we have no thought of attempting an extended analytical notice of this work, but will make a few extracts which will serve as specimens of Dr. Elliotson's very agreeable manner, and the first shall be from his introduction. He is speaking of medical education, and in referring to the two classes of medical men in England—the *physician*, and the *general practitioner*—and to the objection urged by some to an improvement in education, that it would make the latter equal in knowledge to the former, he introduces the following very agreeable history of his rise in his profession:

"In *me* such narrow views would be the height of baseness, and would be the height of ingratitude; for I hesitate not to avow,—I rejoice in this public opportunity of declaring, that to the general practitioners of England, Scotland, and Ireland, I am indebted for success in my profession. When I commenced my professional career, I determined upon seeking for success by working hard; and by conducting myself as well as the infirmity of human nature would allow. I determined, however long I might wait for success, never to fawn upon and run after my superiors, nor to stoop meanly to my inferiors; never to intrigue for an advantage, nor to employ trumpery artifices for making myself known to the public. For many years I toiled; and saw most of my contemporaries, —nay, of my juniors (who worked less, but were wiser in their generation) pass by me. I published work after work, —edition after edition; and paper after paper was honored with a place in the Transactions of the first Medical Society in Europe. I was physician to a large metropolitan hospital; and had attended there, and gratuitously out of doors, above 20,000 patients. But in vain. In the year 1828 my profession was no more lucrative to me than in 1818, and was as short of my actual expenses. At that time the "Lancet" was pleased, now and then, to publish a clinical lecture, delivered by me at St. Thomas's; and my practice at once doubled.

The following year it published the greater part as I delivered them; and my practice doubled again. Next season, the "Lancet" published them all; the "Medical Gazette" followed its example; and my practice doubled a third time. This astonished me the more, as my clinical lectures were generally delivered with little or no premeditation; while all that I published myself had cost me great labor, many a headache, and much "midnight-oil." It was through the general practitioners, in the large majority of instances, and through general practitioners (for the most part) with whom I had not the honor of any acquaintance, that the publication of those lectures accomplished my success. To the body of general practitioners, therefore, I owe a debt of gratitude. They have called me forth spontaneously, from no interested motive; and I cannot exert myself too much in the education of their successors.

The following remarks are just, and are entitled to the careful consideration of the young practitioner:

It is in making an accurate, careful diagnosis, that the medical practitioner chiefly shines. When the nature of a disease is once ascertained, it is no difficult matter to treat it. Other qualities of mind are then required;—frequently only courage, or mere perseverance; but it is in making a diagnosis,—in ascertaining the character of a disease, that the scientific practitioner outshines the inferior. Unfortunately this is a point not attended to as it ought to be. Many persons pride themselves in being good practitioners; because, without knowing what is the matter, they can say what will do good. It is an unscientific way of proceeding; and even if I could not practise better for making a good diagnosis, still I would be as particular on that point as possible, for the sake of observing a good general rule,—for the sake of endeavoring to treat the patient better than I otherwise could, notwithstanding all my conceit; and for the purpose of being ready to meet any unexpected emergency that might arise. A medical man who will not take the trouble to establish a diagnosis, is just like a surgeon who will not condescend to learn the anatomy of hernia; but who says that he knows if he cuts this way and that way, he shall liberate the entangled parts, and accomplish the operation as well as the best. I have witnessed this; but, then, what are such men when a difficulty arises? They are lost in perplexity, and glad to apply to those who know better. It is impossible to be too minute in making a diagnosis.

Of the pulse he remarks:

"We must also remember, as physiologists, that the pulse differs in every age,—that the younger we are, the more quick is the pulse; also that the pulse of the female is quicker than that of the male; and that, generally, the inhabitants of a warm climate have a weaker pulse, than those in a more temperate latitude. It is necessary to take all these things into consideration; because, if we were to find the pulse of a child 120, and that of an adult laboring under fever likewise 120, we should commit a great error in supposing that the child likewise labored under fever. It is necessary, therefore, to remember that the pulse varies according to age, sex, and climate; and that in young children it is particularly quick. I had an instance of the great use of examining a patient in bed, and not being contented without what is contemptuously called "a *mechanical* examination," in an enlargement of the abdomen:—

*Case.*—I was called to visit a pregnant lady, about thirty years of age, whose pulse was about 80 or 90; but on listening to the abdomen,—she being single, and having some disease (as she said,)—there was another pulsation, about 128. The pulsation did not arise from any of the branches of the iliac arteries; for their pulsations would have been the same as at the wrists. She had a pulse of 80; something within her had a pulse of 128; and what that was, I left her to settle by herself. All that I could say was, that if she waited patiently, the whole of the disease would come away, to a certainty, in two or three months."

The author cites many curious cases under the head of "*derangement of the digestive organs*," as follows:

"Sometimes the appetite is not simply *deficient*, but is *depraved.* This is particularly seen in females; and is called "pica" (*a magpie;*—an animal said to be subject to this complaint.) Sometimes young ladies long for chalk, cinders, or sand; and they will bite glass,—munch it; and, when it is small enough, they will swallow some of it. I saw a lady, who ate *brown* paper; she longed for something to eat,—for something that she had never eaten before; but she could not tell what;—nor could I. I have heard of cases where the patient longed for raw flesh, and even for *live* flesh; so that some have eaten live kittens and rats. This is an absolute fact. In the same way, some have been known to long for the contents of snuffers; and even for manure. A case is described of a man who ate a live pig,—leaving the intestines; but, after a little while, he ate them also. There is a case described, at full length, in a German work, of a boy who had such a longing for lime, that he ate all the mortar he could pick out of the wall; and, being well horsewhipped for it, he then commenced on a *neighbor's* wall. In order to prevent this, however, the neighbor smeared the wall with a decoction of wormwood, and the boy could no longer relish it; but he then went to the kennel in the street, and sucked up the sand. He had a desire for something dirty. After this he got to some quick lime; and was forced to drink a large quantity of water, to extinguish his thirst. The mucous membrane within, had a distaste for what is called "*good* food;" but, in other respects, he was quite well. I recollect having read of a girl, and also of a student at Leyden, who always ate spiders when they could get them; and no harm arose from it. I have read of a man who disliked bread, and never ate it; but he was seized with a quartan ague, and then ate a large quantity. He recovered from the ague; and the disgust towards bread returned. This disease occurs in young women (even moral ones) who do not menstruate well;—as I mentioned when speaking of chlorosis; and many pregnant women have some strange longing of this description. Every one must have met with instances of women longing for what it was difficult to get;—longing for things out of season. There was one who longed for a bit of the priest's sleeve, and contrived to get at it and bite it;—not caring for his excommunication. I could relate cases, almost without end, of this description. One dipped her bread in a tar-tub. I never met with these extreme cases; but every one must have read of instances of this nature."

We saw a striking instance of the sort last summer. The subject was a young married woman. She had an ungovernable propensity to eat newspapers: the smell of a printing office nearly distracted her. She ate newspapers every day in large quantities. Her digestion became greatly deranged; bowels torpid; complexion exsanguineous. The depraved appetite was corrected by the use of iron—the prussiate or carbonate—and she is now in pretty good health. Whenever she feels any return of the odd propensity, she resorts to the iron, which she has found to be an effectual remedy against it.

Concerning sluggishness of the bowels, our author has the following observations:

"Very frequently, the stomach is not in fualt; but there is a sluggishness of the intestines; and, as the cause is situated there, a regular course of purgative medicine will be found absolutely necessary. It is wrong to give *strong* purgatives. Those which open the bowels regularly, are best adapted for the purpose. It is astonishing how long things taken into the stomach and intestines, will remain quiet; and then give rise to various symptoms. I alluded to the circumstance before. In the "Philadelphia Journal," for 1822, there is an account of a coagulum of milk, which was vomited two months after it had been taken. I have myself seen a coagulum of milk, like bird-lime; which had remained some days (even a week) in the patient's stomach, and produced the greatest uneasiness;—the stomach not being in fault, but being oppressed by this particular substance. I have seen a piece of salmon vomited by an infant, a month after the nurse had been so foolish as to give the child that food. I mentioned, that Dr. Barlow (of Bath) published a case, in which pills of sulphate of iron were discharged, *per anum*, nine months after they had been taken. In a foreign journal, a case is mentioned, similar to that of Dr. Barlow's;—a case in which pills were vomited, a year after they were taken. An instance is recorded, in which a blacksmith's son bolted thirty grapes. He did not *masticate*, but swallowed them *whole;* and, after three months frequent vomiting and extreme suffering, he was cured by an active purgative medicine. Ten of the grapes came away whole, even then. A case is mentioned by Bartholini, where a coagulum of milk, as large as a man's tongue, was discharged, after the patient had taken muriatic acid. The same author also mentions a case, where a patient had swallowed a swine's tooth. He suffered under hypochondriasis and extreme emaciation, for two years; the tooth was then discharged *per anum;* and the individual perfectly recovered. Occasionally, then, we may have disorder of the digestive organs from articles which have been taken, and have remained in the stomach and intestines a much longer time than we could imagine. If, in a case of disorder of the digestive organs, we suspect any thing of this kind, we ought to employ strong remedies. Purgatives and injections, of course, are very important.

"But whether this be the cause of the disease or not, it is of the highest importance to keep the bowels regular. Nothing can be worse than to give strong purgutives for this purpose; except where there is something considerable to be brought away. In a case of habitual costiveness, strong purgatives are decidedly bad; because, when we have once acted violently on the intestines, the latter, according to the laws of nature, must fall into a torpid state,—go to sleep. During this condition of repose, the fæces accumulate again; so that we have to give another strong dose, in order to remedy the mischief of the first. Thus the person is always costive, or taking strong medicines; the consequence of which is, that he is at last obliged to take them, or he will fall into a state of dyspepsia."

Our readers would hardly pardon us if we closed this article without some further account of the American editor, in whose history they must, by this time, feel an extraordinary interest. Be it known to them, then, that he was educated in Paris, under the fostering care of Louis and Chomel, whom he appears to regard himself as having already distanced in his scientific achievements. He is the translator of a memoir on Emphysema of the lungs, by one of his illustrious Parisian masters, M. Louis, physician to La Pitié. In this paper he takes occasion to say, that having had an opportunity, during his daily visits to that establishment, of observing for *himself* a portion of the patients referred to by the author, he is able to *vouch for the care and attention bestowed upon their examination.* Notwithstanding all this care, such was his astonishing acuteness that he was able to discover and correct several errors contained in the original—a circumstance which, if we consider the infallibility of the French observers, must be allowed to place him in the very front rank of medical philosophers.

Dr. Stewardson is also a contributor to the pages of the American Journal of the Medical sciences. His papers, when the writings of others have been under consideration, are marked by a singular spirit of ingenuousness. He is scrupulous never to distort the meaning of the author, and rarely misquotes a sentence, where he supposes there is much danger of detection. If his style is not always the most courteous, it is plainly because there are few men, living or dead, whose writings he considers as entitled to the slightest respect. An author of so much genius can hardly be expected to regard the efforts of the common race of mortals, with any other feelings than those of contempt. His acerbity of manner, and his numerous misrepresentations must be ascribed to this genius, which is too proud to look closely enough at the labors of others to understand them.

The most important original papers of our *savan* are contained in the numbers of that journal, for April 1841 and April 1842: they are entitled "Observations on Remittent Fever," and are illustrated by the numerous cases of the disease which the author had an opportunity of witnessing in the Pennsylvania Hospital, of which he is so distinguished an ornament. This paper leaves nothing to be desired,—the subject is literally exhausted; and if the disease which it investigates has not been effectually eradicated from the human system, it is not the fault of its amiable and candid author. To attempt any thing like an analysis of it at this remote period would be a work of supererogation, particularly when it is remembered that the journal in which it originally appeared is read and admired in every part of the world where medicine is cultivated as a science, where profound thought, original research, and correct writing are appreciated, or where the supremacy of genius maintains its elevated and life-diffusing influence.

The latest contributions of our editor are, we believe, his "Notes and Additions" to the work, the title of which stands at the head of the present article. These consist mainly of two chapters on remittent and yellow fevers, topics as he informs us, but cursorily treated by Dr. Elliotson and his other commentators.

We were disposed to find fault with Dr. Stewardson for the brevity of his notes and editions, until we recollected that the intrinsic value of a work does not always consist in the number or magnitude of its pages. In composing the two articles under consideration, the author was evidently governed by the maxim of the ancient philosopher, that "a little thing gives perfection, though perfection is not a little thing." The chapter on remittent fever is condensed from the immortal paper, already adverted to; that on yellow fever appears for the first time in these pages. It occupies precisely *eight pages and a third*, and is founded upon the observation of *a case* of that disease as it occurred in the Pennsylvania hospital. In this short space we are presented with a most graphic outline of the natural history of this truly horrible malady, its anatomical characters, diagnosis and nature, causes, mortality and other circumstances, together with a most able and learned account of the treatment. Considering his limited opportunities, it must be acknowledged that Dr. Stewardson has done wonders. He has in, truth, afforded full proof of the proposition, that "it is the peculiar privilege of genius to accomplish ends without advantages, and to overcome difficulties insurmountable to ordinary minds." If he had enjoyed more enlarged opportunities of observing and studying yellow fever, there is no doubt whatever that he would have exhausted the subject, as he has that of remittent fever. When we reflect upon his extraordinary powers as a writer and an observer, we cease to wonder that he did not, in this part of his labors, think worth while to avail himself of the researches of Rush, Jackson, Catel, and others—authors whom he esteems as quite obsolete. Louis. as is natural, he mentions with a singular degree of complacency, which arises probably not so much from any supposed excellence, as because it was his good fortune to be the preceptor of Dr. Stewardson. Several other writers, are also occasionally alluded to, but evidently with a degree of suspicion, clearly indicating that very little confidence is to be placed in their productions. This is as it should be; nay, as it must be. They lived before the discovery of the *numerical method*—before Louis, Chomel, and Stewardson, and therefore justly deserve to be forgotten.

Judging from the extraordinary ability displayed in the whole of this chapter on yellow fever, and the circumstance that it is founded upon the observation of a *solitary case of the*

*disease*, we are convinced that Dr. Stewardson is the only man in the profession competent to furnish a treatise on the art of writing medical books. We would therefore respectfully suggest to him the propriety of such an effort.

The modesty of Dr. Stewardson—we must be pardoned for recurring to this amiable trait in his character—is strikingly exhibited in the position he has taken for his additions to Elliotson's Practice. It is well known that the most prominent parts of every book are the beginning and the end thereof. Now, instead of introducing his "two chapters" at one or the other of these points, he has modestly placed them in the middle of "*Stewardson's Elliotson*," where, but for their superior excellence, and the circumstance of their being included in brackets, and headed by the name of the author in large capitals, they might readily escape the eye of the heedless student.

Dr. Stewardson, we are sure, does not belong to the tribe of writers, of whom we have spoken, who fancy themselves at liberty to mutilate the writings of European physicians, and engraft upon them their own crudities. He is a writer of parts, and we cannot forbear to thank him for the opportunity he has afforded us, by his contributions to this work, of introducing him to his backwoods' brethren. His are productions which they "will not willingly let die." Elliotson, President of the Phrenological Society of London, and late Professor of the Principles and Practice of Medicine in University-College, might have been forgotten; and even the name of Lord Brougham might have passed from the memories of men, but for its fortunate association with that of Thomas Stewardson. They are indeed lucky men—

O fortunatos nimium sua si bona norint.

